# Cannabinoid receptor type 2 activation induces a microglial anti-inflammatory phenotype and reduces migration via MKP induction and ERK dephosphorylation

**DOI:** 10.1186/1744-8069-5-25

**Published:** 2009-05-28

**Authors:** Edgar Alfonso Romero-Sandoval, Ryan Horvath, Russell P Landry, Joyce A DeLeo

**Affiliations:** 1 Department of Anesthesiology, Neuroscience Center at Dartmouth, Dartmouth Medical School, Dartmouth College, 1 Medical Center drive, Lebanon, New Hampshire 03756-1000, USA; 2 Department of Pharmacology and Toxicology, Dartmouth Medical School, Hanover, New Hampshire 03755, USA

## Abstract

**Background:**

Cannabinoid receptor type 2 (CBR2) inhibits microglial reactivity through a molecular mechanism yet to be elucidated. We hypothesized that CBR2 activation induces an anti-inflammatory phenotype in microglia by inhibiting extracellular signal-regulated kinase (ERK) pathway, via mitogen-activated protein kinase-phosphatase (MKP) induction. MKPs regulate mitogen activated protein kinases, but their role in the modulation of microglial phenotype is not fully understood.

**Results:**

JWH015 (a CBR2 agonist) increased MKP-1 and MKP-3 expression, which in turn reduced p-ERK1/2 in LPS-stimulated primary microglia. These effects resulted in a significant reduction of tumor necrosis factor-α (TNF) expression and microglial migration. We confirmed the causative link of these findings by using MKP inhibitors. We found that the selective inhibition of MKP-1 by Ro-31-8220 and PSI2106, did not affect p-ERK expression in LPS+JWH015-treated microglia. However, the inhibition of both MKP-1 and MKP-3 by triptolide induced an increase in p-ERK expression and in microglial migration using LPS+JWH015-treated microglia.

**Conclusion:**

Our results uncover a cellular microglial pathway triggered by CBR2 activation. These data suggest that the reduction of pro-inflammatory factors and microglial migration via MKP-3 induction is part of the mechanism of action of CBR2 agonists. These findings may have clinical implications for further drug development.

## Background

Microglia are the innate immune cells of the central nervous system (CNS) and as such act as the first glial responders after CNS or peripheral nerve injury [1-3]. The main responses of microglia to peripheral or CNS insults are increased expression of surface or cytosolic markers, pro-inflammatory factor production (e.g. cytokines, chemokines, nitric oxide, prostaglandins), morphological changes, enhanced phagocytic activity, migration and proliferation. In rodent models of pain including peripheral nerve injury [4], paw incision [5], paw inflammation [6] or spinal cord injury [7], microglia become reactive and produce a pro-inflammatory spinal milieu, which may contribute to neuronal sensitization and behavioral hypersensitivity.

Cannabinoids exert most of their effects by binding to G protein-coupled cannabinoid receptors (CBR) type 1 and 2. CBR2 are expressed in glia in normal human and rat brain [8,9] and their glial expression increases especially during inflammation [10,11]. Using a rat paw incision or a peripheral nerve injury model we have previously shown that *in vivo *spinal CBR2 activation reduces glial reactivity, measured as a reduction in the expression of CR3/CD11b or ionized calcium-binding adaptor molecule 1 (Iba-1) in microglia [12,13]. Iba1 is a cytosolic microglial marker that is associated with a pro-inflammatory phenotype and is involved in microglial migration [14,15]. Accordingly, *in vitro *CBR2 activation reduces tumor necrosis factor-α (TNF) and nitric oxide (NO) production in primary microglia [11,16] and is protective against neurotoxicity of human microglia [17]. Nonetheless, the specific intracellular mechanism of action by which CBR2 activation alters the microglial phenotype has not been previously reported.

Microglial p-ERK plays a central role in the mechanisms underlying spinal cord injury-, nerve injury- and diabetes-induced hypersensitivity [7,18-20]. Microglial p-ERK inhibition reduces TNF production [21]. In addition, spinal TNF blockade reduces peripheral nerve injury-induced allodynia [22]. Cell migration is mediated by p-ERK [23,24]. However, the role of p-ERK in microglial migration is not known. We hypothesized that CBR2 activation reduces microglial p-ERK, and subsequently TNF production and cell migration.

Mitogen-activated protein kinase-phosphatases (MKP) regulate several pro-inflammatory pathways and display distinct substrate preferences for various mitogen-activated protein kinases (MAPKs) [25]. For example, MKP-3 is a selective ERK pathway negative regulator [26,27] and MKP-1 mainly down-regulates p38 or JNK [28], but may regulate ERK [29]. The role of phosphatases in microglial inflammatory processes has yet to be clarified. Therefore, we also hypothesized that microglial CBR2 activation reduces p-ERK by inducing MKP-1 and MKP-3. Herein, we study a specific signaling pathway in primary microglia to elucidate the molecular mechanisms of action of CBR2 activation.

## Results

### Microglial CBR2 activation induces MKP-1/3 and reduces p-ERK and TNF

**Figure 1:**
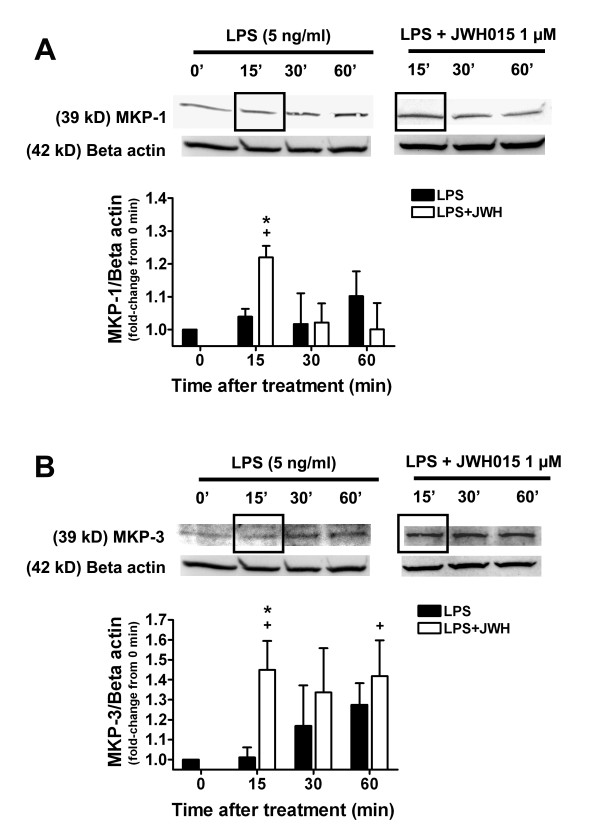
**Effects of microglial CBR2 activation on the MKP-1/3 pathway**. Representative western blots and quantification of MKP-1 (A) and MKP-3 expression (B) at different incubation time points (15–60 min, 0 = medium control group) in LPS-stimulated microglia in the presence or absence of JWH015 (1 μM). JWH015 produced a MKP-1 and MKP-3 increase (15 min). +p < 0.05 vs. medium control group; *p < 0.05 vs. LPS alone group.

First, we determined the effects of JWH015 on MKP-1 and MKP-3 expression in LPS-stimulated microglia. LPS did not significantly change the levels of MKP-1 expression compared to the medium control group at the tested time points (15–60 min, Figures 1A). However, MKP-1 expression was significantly increased in LPS + JWH015 only at 15 min incubation time point compared to the 0 time point (the medium control group, 1.22 ± 0.04 of medium control group, p < 0.05; Figures 1A). This increased MKP-1 expression in LPS + JWH015 group was also significantly different from the LPS alone group at the same time point (15 min, 1.22 ± 0.04 vs. 1.04 ± 0.02 of medium control group respectively, p < 0.05, Figures 1A). LPS did not significantly change the levels of MKP-3 expression compared to the medium control group at the tested time points (Figures 1B). MKP-3 expression was significantly increased in LPS + JWH015 at 15 and 60 min incubation time points (1.45 ± 0.14 and 1.42 ± 18 of medium control group respectively, p < 0.05; Figures 1B). This increased MKP-3 expression in LPS + JWH015 group was also significantly different from the LPS alone group at the 15 min incubation time point (15 min, 1.45 ± 0.14 vs. 1 ± 0.07 of medium control group respectively, p < 0.05, Figures 1B).

MKP-1 has been shown to be inducible (for example by LPS) but not constitutively expressed in macrophages [30]. To test whether MKP-1 and MKP-3 are constitutively expressed in microglial cells we incubated primary microglia in serum free medium (DMEM) or in DMEM plus 10% fetal bovine serum (S+DMEM) for 1.5 or 20 h. We observed that primary microglial cells constitutively expressed MKP-1 and we found no differences in the DMEM and S+DMEM groups or 1.5 and 20 h incubation periods (1.5 hr DMEM vs. S+DMEM = 0.97 ± 0.1 vs. 0.98 ± 0.2; 20 hr DMEM vs. S+DMEM = 0.75 ± 0.02 vs. 0.72 ± 0.02). We also confirmed a constitutive expression of MKP-3 in microglia with no differences between DMEM and S+DMEM group or 1.5 and 20 h incubation periods (1.5 hr DMEM vs. S+DMEM = 1.3 ± 0.04 vs. 1.4 ± 0.3; 20 hr DMEM vs. S+DMEM = 1.1 ± 0.03 vs. 1.1 ± 0.02). These results also suggest that our microglial cells are in a quiescent rather than a primed state prior to our treatments.

**Figure 2:**
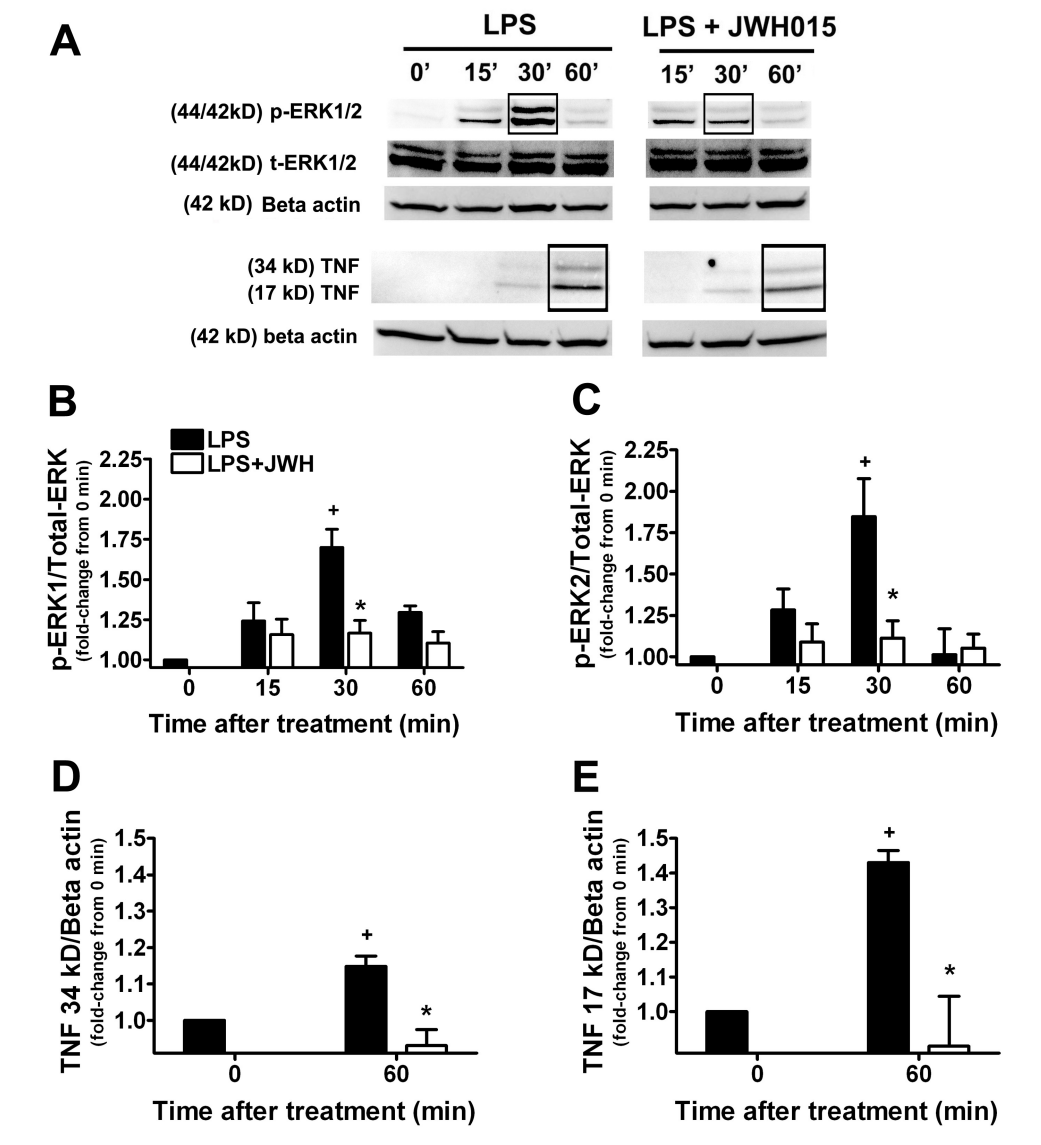
**Effects of microglial CBR2 activation on the ERK/TNF pathway**. Representative western blots (A) and quantification of p-ERK1 (B), p-ERK2 (C) and TNF (D, 34 kD; E, 17 kD) expression at different incubation time points (15–60 min, 0 = medium control group) in LPS-stimulated microglia in the presence or absence of JWH015 (1 μM). JWH015 produced a ERK dephosphorylation (30 min) and TNF reduction (60 min). +p < 0.05 vs. medium control group; *p < 0.05 vs. LPS alone group.

MKP-1 decreases MAPKs, such as p38, c-Jun N-terminal kinase (JNK) or ERK1/2 [28,29]. MKP-3 is a specific and major negative regulators of the ERK pathway [25,26]. We investigated the functional implications of MKP-1 and MKP-3 modulation by studying ERK1/2 phosphorylation. LPS did not induce any significant change in t-ERK1/2 at any time point studied and JWH015 did not modify this (Figure 2A). The expression of t-ERK1/2 was not significantly different at any incubation time point in the LPS + JWH015 group compared to the medium control group, except at the 120 min time point in LPS + JWH015 compared to medium control group (Figure 2A). No differences were found in t-ERK1/2 expression between LPS alone and LPS + JWH015 groups in any of the incubation time points (Figure 2A).

LPS induced a significant increase in p-ERK1 expression at 30 min (1.7 ± 0.11 of medium control group, p < 0.05; Figures 2A and 2B), and in p-ERK2 at the 30 min incubation time point compared to the medium control group (1.85 ± 0.23 of medium control group, p < 0.05; Figures 2A and 2C). The expression of p-ERK1/2 was not significantly different in the LPS + JWH015 group compared to the medium control group at any of the incubation time points. Moreover, p-ERK1/2 expression in LPS+JWH015 was significantly reduced at the 30 min incubation time point compared to the LPS alone group (p-ERK1 = 1.7 ± 0.11 vs. 1.17 ± 0.08 of medium control group respectively, p-ERK2 = 1.85 ± 0.23 vs. 1.11 ± 0.1 of medium control group respectively, p < 0.05; Figures 2A, B and 2C).

ERK mediates the LPS-induced microglial pro-inflammatory factor, TNF [21,31,32]. We further investigated the functional implications of p-ERK modulation by studying TNF expression. LPS induced an increase in TNF (17 kD [monomer] and 34 kD [dimer]) expression at the 60 min incubation time point compared to the medium control (34 kD = 1.15 ± 0.03, and 17 kD = 1.43 ± 0.03 of medium control group, p < 0.05, Figures 2A, D and 2E). TNF expression was not significantly different between the LPS + JWH015 group compared to the medium control group. Furthermore, 34 kD TNF expression was significantly reduced in LPS + JWH015 group compared to the LPS alone group at the 60 and 120 min incubation time points (0.93 ± 0.04 vs. 1.14 ± 0.3 and 0.91 ± 0.05 vs. 1.05 ± 0.1 of medium control group 60 and 120 min respectively, p < 0.05; Figures 2A and 2D) and 17 kD TNF expression was significantly reduced in LPS + JWH015 group compared to the LPS alone group at the 60 min incubation time point (0.90 ± 0.14 vs. 1.43 ± 0.03 of medium control group 60 min, p < 0.05; Figure 2A and 2E). We confirmed the dependence of TNF expression on ERK phosphorylation in LPS-stimulated microglia by inhibiting MAP/ERK kinase (MEK) with UO126 (1 μM). TNF expression was reduced in the LPS + UO126 group compared to the LPS alone group at the 60 min incubation time point (40.6 ± 12.1% inhibition, p < 0.05, data not shown). UO126 completely inhibited p-ERK in the LPS + UO126 group compared to the LPS alone group at the 30 and 60 min incubation time points (data not shown).

### MKP-3 inhibition induces microglial p-ERK in LPS+JWH015-treated cells

**Figure 3:**
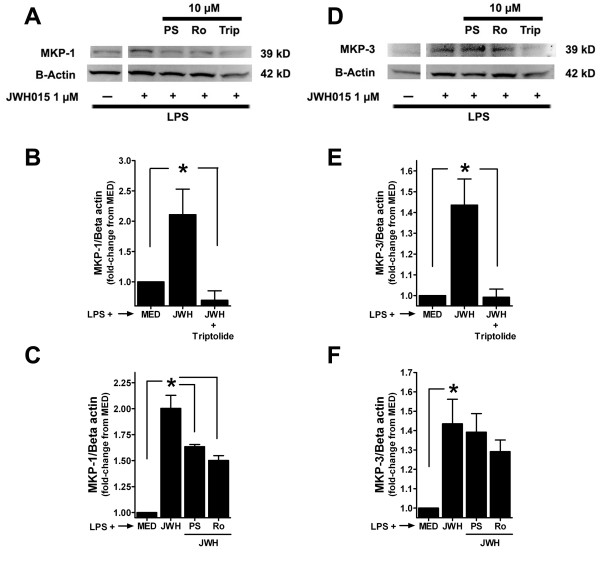
**Blockade of JWH015's effects on MKP-1 and MKP-3**. (A) Representative western blots of MKP-1 and beta-actin in primary microglia treated with LPS in the absence or presence of JWH015 (15 min incubation, JWH). Cells were pre-incubated with or without 10 μM PSI2106 (PS), Ro-31-8220 (Ro) or triptolide (Trip) for 30 min. (B) Triptolide (10 μM) completely blocked the MKP-1 expression induced by JWH015 (1 μM). (C) PSI2106 or Ro-31-8220 partially inhibited the MKP-1 expression induced by JWH015. (D) Representative western blots of MKP-3 and beta-actin in primary microglia treated with LPS in the absence of presence of JWH015 (15 min incubation). Cells were pre-incubated with or without PSI2106, Ro-31-8220 or triptolide (10 μM) for 30 min. (E) Triptolide (10 μM) completely blocked the MKP-3 expression induced by JWH015 (1 μM). (F) PSI2106 or Ro-31-8220 did not modify the MKP-3 expression induced by JWH015. *p < 0.05 vs. LPS + medium (MED) or LPS + JWH015 + PSI2106, Ro-31-8220 or triptolide group (indicated by the connecting lines).

In these studies we challenged JWH015's effects on MKP-1 and MKP-3 using three different compounds claimed to be MKP-1 inhibitors. Cells were pre-incubated with or without PSI2106, Ro-31-8220 or triptolide (1 and 10 μM) for 30 min, then LPS with or without JWH015 (1 μM) was added for 15 min. The expression of both MKP-1 and MKP-3 were significantly enhanced in LPS+JWH015 group (p < 0.05) as shown previously. MKP-1 expression induced by JWH015 in LPS-stimulated microglia was not modified by 1 μM PSI2106 or Ro-31-8220 (2 ± 0.13 vs. 1.95 ± 0.02 and 2.02 ± 0.3 of medium control, LPS+JWH015 vs. LPS+JWH015+PSI2106 or Ro-31-8220 respectively), but it was inhibited following treatment with the dose of 10 μM (2 ± 0.13 vs. 1.6 ± 0.02 and 1.5 ± 0.05 of medium control, LPS+JWH015 vs. LPS+JWH015+PSI2106 or Ro-31-8220 respectively, p < 0.05, Figures 3A and 3C). PSI2106 or Ro-31-8220 did not modify JWH015's effects on MKP-3 at 1 μM (1.43 ± 0.13 vs 1.58 ± 0.09 and 1.64 ± 0.16 of medium control, LPS+JWH015 vs. LPS+JWH015+PSI2106 or Ro-31-8220 respectively) or 10 μM concentration (Figures 3D and 3F). However, triptolide (only at 10 μM) completely inhibited the JWH015-induced MKP-1 (Figures 3A and 3B) and MKP-3 (Figures 3D and 3E) expression in LPS-stimulated microglia. Triptolide at 1 μM did not inhibit JWH015-induced MKP-1 (2 ± 0.13 vs. 1.93 ± 0.04 of medium control, LPS+JWH015 vs. LPS+JWH015+triptolide respectively) or MKP-3 (1.43 ± 0.13 vs 1.55 ± 0.17 of medium control, LPS+JWH015 vs. LPS+JWH015+triptolide respectively) in LPS-treated microglia.

**Figure 4:**
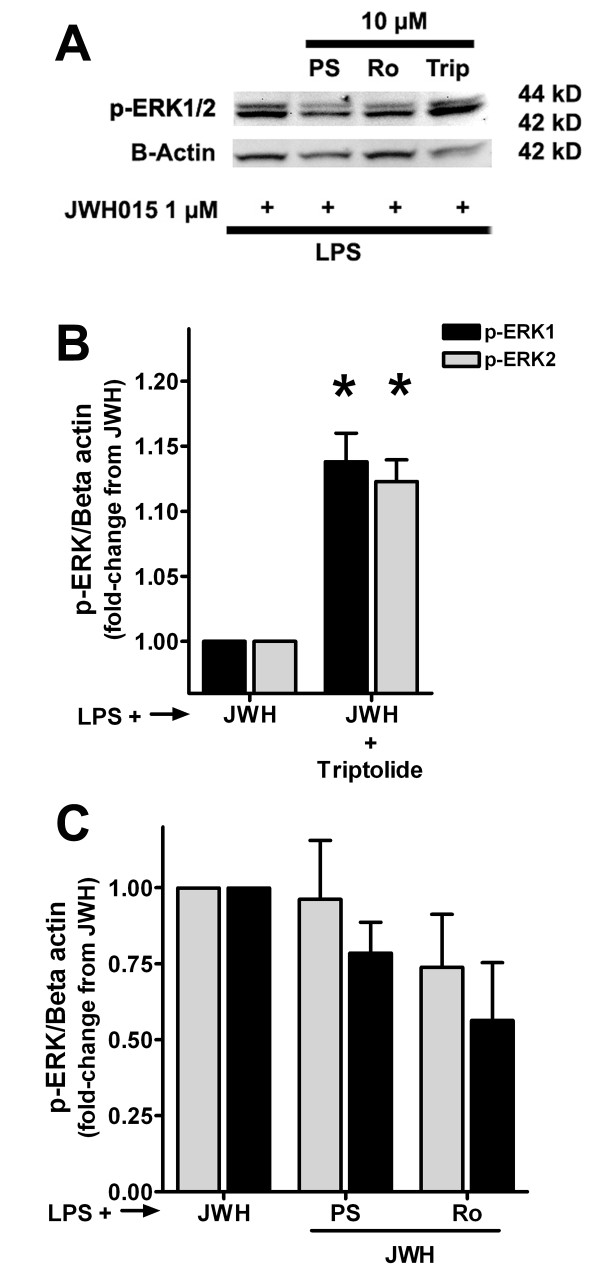
**Effects of MKP-1 and/or MKP-3 inhibition on p-ERK1/2**. (A) Representative western blots of p-ERK1/2 and beta-actin in primary microglia treated with LPS and JWH015 (15 min incubation, JWH). Cells were pre-incubated with or without 10 μM PSI2106 (PS), Ro-31-8220 (Ro) or triptolide (Trip) for 30 min. (B) Triptolide increased the expression of p-ERK1/2 in parallel with the inhibition of both MKP-1 and MKP-3. C. PSI2106 or Ro-31-8220 did not modify p-ERK1/2 expression. *p < 0.05 vs. LPS + JWH015.

**Figure 5:**
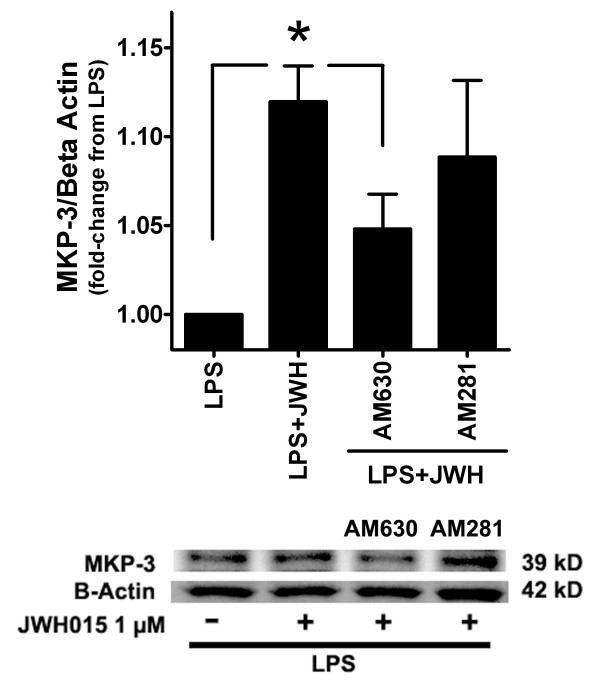
**Blockade of CBR2 antagonizes the MKP-3 induction by JWH015**. Quantification and representative western blot of MKP-3 expression in LPS-stimulated microglia in the presence or absence of JWH015 (1 μM) with or without the CBR2 antagonist, AM630, or the CBR1 antagonist, AM281. JWH015 induced a significant increase in MKP-3 compared to the LPS alone groups at the 15 min incubation time point. However, AM630 significantly blocked JWH015's effects on MKP-3 expression. JWH015's effects on MKP-3 were not modified by AM281. *p < 0.05 vs. LPS alone and LPS + JWH015 1 μM groups.

We evaluated the expression of p-ERK1/2 in the above mentioned groups to confirm the functional implications of the inhibition of MKP-1 and/or MKP-3. Triptolide (10 μM), which completely inhibited the induction of both MKP-1 and MKP-3 by JWH015, induced a significant increase in p-ERK1 (1.14 ± 0.02 of LPS + JWH015 control group, p < 0.05; Figures 4A and 4B) and p-ERK2 (1.12 ± 0.02 of LPS + JWH015 control group, p < 0.05; Figures 4A and 4B). Triptolide at 1 μM concentration did not significantly modify the expression of p-ERK1 (1.11 ± 0.06 of LPS + JWH015 control group) or p-ERK2 (1.09 ± 0.08 of LPS + JWH015 control group) in LPS plus JWH015-treated cells. Unexpectedly, neither PSI2106 nor Ro-31-8220 (which effectively blocked only the JWH015-induced MKP-1 but not MKP-3 expression) modified p-ERK1 (1 μM: 1.04 ± 0.05 and 0.99 ± 0.06 of LPS + JWH015 control group, LPS+JWH015+PSI2106 or Ro-31-8220 respectively; Figure 4A and 4C) or p-ERK2 (1.03 ± 0.06 and 0.99 ± 0.02 of LPS + JWH015 control group, LPS+JWH015+PSI2106 or Ro-31-8220 respectively; Figure 4A and 4C). These data together suggest that the induction of MKP-3 rather than MKP-1 is the mechanism by which JWH015 reduces p-ERK1/2 expression. LPS plus triptolide (10 μM) in the absence of JWH015, did not modify the expression of MKP-1, MKP-3, t-ERK and p-ERK when compared to the LPS alone group (data not shown). Since our results suggest that MKP-3 rather than MKP-1 is responsible for the p-ERK1/2 reduction induced by JWH015, we challenged the effects of JWH015 on MKP-3 expression with specific cannabinoid antagonists. The selective CBR2 antagonist, AM630, but not the CBR1 antagonist, AM281, significantly blocked the JWH015-induced MKP-3 in LPS stimulated microglia (1.12 ± 0.02 vs. 1.05 ± 0.02 of LPS alone group, LPS + JWH015 1 μM in absence or presence of AM630 1 μM respectively, p < 0.05; Figure 5).

### CBR2 activation and p-ERK inhibition reduce microglial migration

**Figure 6:**
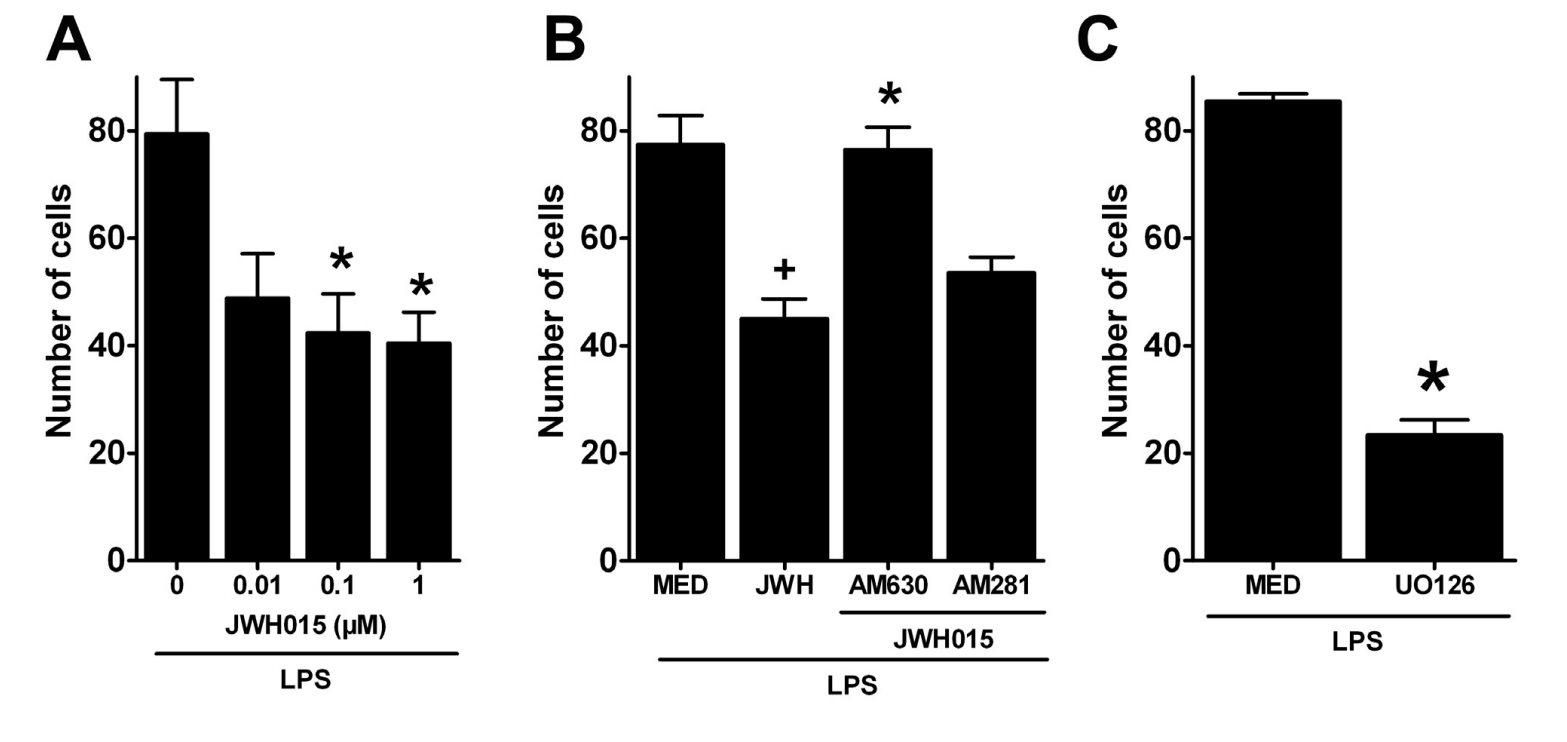
**Quantification of JWH015-reduced LPS-stimulated microglial migration**. (A) JWH015 reduced the number of LPS-stimulated microglia that migrated towards ADP in a dose related fashion. *p < 0.05 vs. medium control group (0) group. (B) Blockade of the effects of JWH015 (1 μM,) on microglial migration by the selective CBR2 antagonist, AM630, but not by the selective CBR1 antagonist, AM281. Data are presented as the number of cells that migrated towards ADP. +p < 0.05 vs. medium group (MED), *p < 0.05 vs. JWH015 1 μM group (JWH) group. (C) Blockade of microglial migration by the selective MEK inhibitor UO126 in LPS-stimulated microglia. UO126 (1 μM), which selectively blocked MEK and thereby ERK phosphorylation, significantly reduced the number of LPS-stimulated microglia that migrated towards ADP. *p < 0.05 vs.medium group (MED) group.

We observed that JWH015 (1 μM) does not induce microglial chemotaxis (compared to medium as chemoattractant) in LPS-stimulated microglia (data not shown). To determine the effects of JWH015 on microglial migration, we performed a JWH015 dose-response pre-treatment experiments using LPS-stimulated microglia and ADP 10 μM as chemoattractant. JWH015 reduced the ADP-induced microglial migration in a significant and dose related fashion. The numbers of migrated LPS-stimulated cells in JWH015 0.1 and 1 μM groups were significantly lower compared to the LPS alone group (79 ± 10 cells in LPS alone group vs. 42 ± 7 and 40 ± 6 cells in LPS+JWH015 0.1 and 1 μM respectively, p < 0.05; Figure 6A). The effect observed in LPS+JWH015 1 μM group was blocked by the CBR2 selective antagonist AM630, but not by the CBR1 selective antagonist AM281 (45 ± 3 vs. 73 ± 4 or 54 ± 3 cells in LPS+JWH015+AM630 or AM281 respectively; Figure 6B). We have previously shown that minocycline (60 μM) reduced cell migration towards ADP (10 μM) using non-stimulated primary microglia [33]. Herein, we used minocycline as a positive control for our cell migration experiments using LPS-stimulated primary microglia. We observed that minocycline (60 μM) significantly reduced the migration of LPS-stimulated microglia towards ADP (85 ± 4 vs. 24 ± 4 cells LPS alone and LPS plus minocycline groups respectively, p < 0.05, data not shown).

Cell migration is a p-ERK dependent phenomenon [23,24] that to our knowledge, has not yet been studied in LPS-activated primary microglia. We studied the effects of a selective MEK inhibitor, UO126, on LPS-stimulated microglial migration. The inhibition of MEK and subsequently inhibition of ERK phosphorylation with UO126 reduced in a significant manner the number of LPS-stimulated microglial cells that migrated towards ADP (77 ± 11 vs. 23 ± 7 cells, LPS alone and LPS + UO126 groups respectively, p < 0.05; Figure 6C). Together these data confirm that p-ERK1/2 regulates LPS-stimulated microglial migration and suggest that JWH015's effects on LPS-stimulated microglial cells are due to MKP-1/3 induction, and subsequent ERK dephosphorylation.

### MKP-1/3 inhibition reverses CBR2-induced microglial migration inhibition

**Figure 7:**
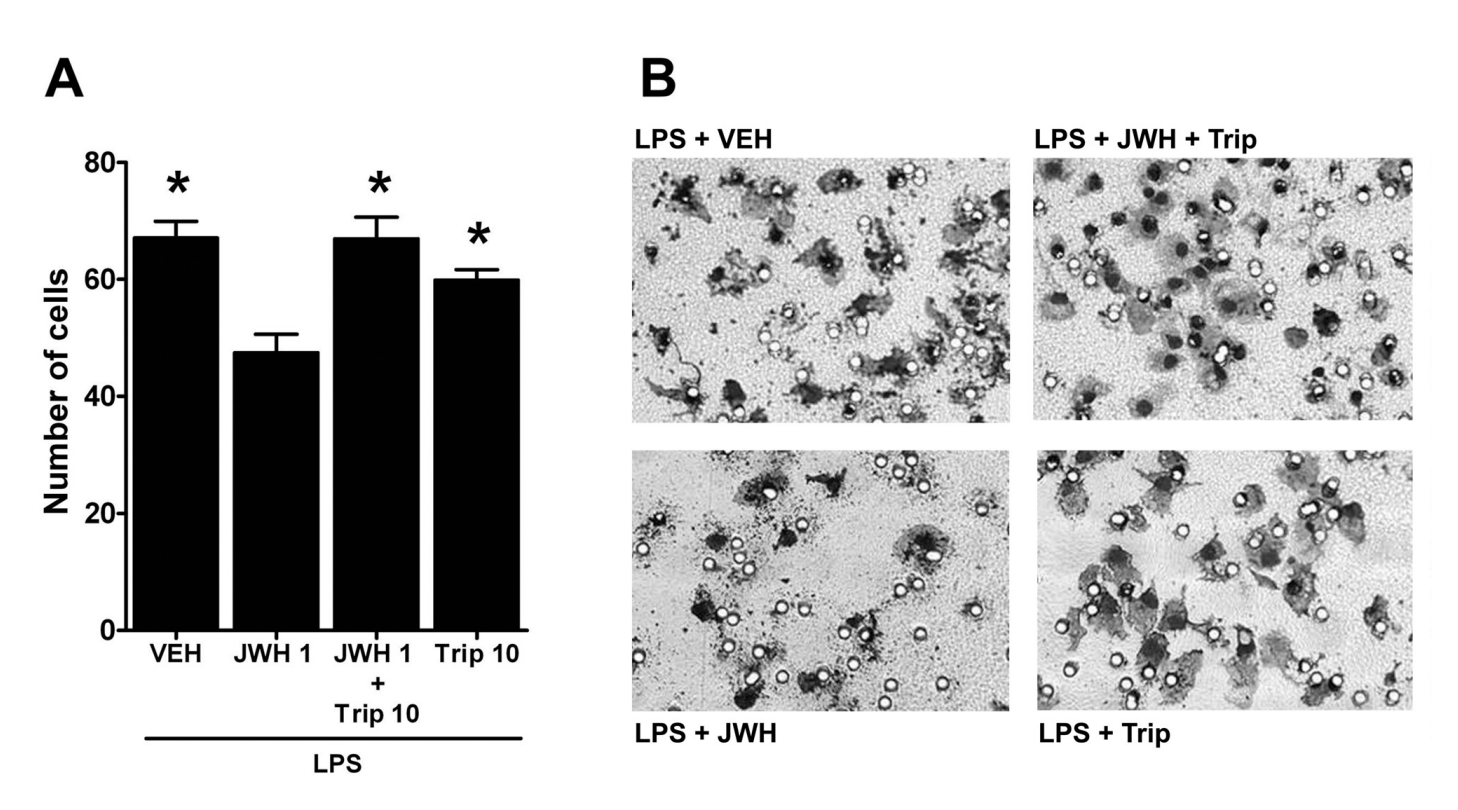
**Blockade of the JWH015 effects in microglial migration by MKP-1 and MKP-3 inhibition**. (A) JWH015 1 μM (JWH 1) significantly reduced the number of LPS-stimulated microglia that migrated towards ADP and triptolide 10 μM (Trip 10) blocked this effect. Triptolide 10 μM did not modify the number of LPS-stimulated microglia that migrated towards ADP. *p < 0.05 vs. JWH015 group. (B) Representative photomicrographs of the migration chamber membranes with attached microglial cells stained with crystal violet. Cells (black) migrated through the pores (white circles) and towards ADP placed into the lower well.

The following experiments were designed to test the functional implications of JWH015 effects on MKP-1/3 expression (which results in ERK dephosphorylation) and LPS-stimulated microglial migration. JWH015-reduced microglial migration was challenged with triptolide 10 μM, which completely blocked JWH015's effects on microglial MKP-1 and MKP-3 expression. The number of cells that migrated towards ADP was significantly lower in the LPS+JWH015 group compared to the LPS alone group (47 ± 3 vs. 67 ± 3 cells respectively, p < 0.05, Figures 7A and 7B). This effect of JWH015 was significantly inhibited by triptolide (67 ± 4 cells compared to LPS + JWH015, p < 0.05; Figures 7A and 7B). Triptolide in the presence of LPS did not affect microglial migration compared to the LPS alone control group (60 ± 2 cells, Figures 7A and 7B).

## Discussion

We demonstrated that selective CBR2 activation results in a change from a pro- to an anti-inflammatory microglial phenotype by increasing the expression of MKP-3. We further showed the functionality of this phosphatase modulation and microglial anti-inflammatory phenotype by demonstrating that JWH015-induced MKP-3 inhibited the ERK pathway, which in turn reduced TNF expression and microglial migration towards ADP. Together, these data support a novel mechanism of action of CBR2 agonists via MKP-3 that may explain their anti-inflammatory, anti-allodynic and/or glial modulatory effects *in vivo*.

Our results showed that JWH015-induced ERK dephosphorylation depends on a MKP-3 increase, rather than MKP-1. Triptolide, which blocked both MKP-1 and MKP-3 induction by JWH015, increased p-ERK in parallel. Triptolide alone has been shown to reduce inflammatory factors in LPS- or amyloid-β1–42-stimulated microglia [34,35]. However, in our setting triptolide alone did not modify the expression of MKP-1/3, t-ERK and p-ERK in the presence of LPS. Triptolide is an effective MKP-1 inhibitor, and therefore a blocker of pharmacological anti-inflammatory effects in LPS-stimulated microglia and monocytes [28,36,37]. We extended these findings by showing that triptolide is also a MKP-3 inhibitor. Interestingly, the blockade of JWH015-induced MKP-1 by PSI2106 or Ro-31-822 did not affect p-ERK. Similarly, other anti-inflammatory drugs, such as dexamethasone induce MKP-1 and, in turn, dephosphorylate p38 and JNK without affecting p-ERK in LPS-stimulated primary microglia [28]. MKPs display distinct substrate preferences for various MAPKs [25]. In the present study, we showed that MKP-3 selectively targets the ERK pathway in microglia, as demonstrated previously in *in vitro *and *in vivo *human skin fibroblasts, or during chick, mouse and zebrafish limb/fin development [25,26]. The fact that MKP-3 is pivotal to reduce p-ERK makes MKP-3 an attractive target for drug development.

Microglial p-ERK plays a key role in neuropathic pain [18], but it is unknown by which specific mechanisms. Additionally, the intracellular molecular mechanisms and cellular functional effects from p-ERK modulation are not fully understood. Herein, we demonstrated that p-ERK is necessary for microglial TNF expression and migration. We confirmed that microglial CBR2 activation reduces TNF expression [11,16] and ERK dephosphorylation results in a significant reduction of TNF in primary microglia [21]. Multiple pathways are involved in the production of this pro-inflammatory cytokine, but our results show a time dependent effect of CBR2 activation on MKP induction (15 min incubation), ERK dephosphorylation (30 min incubation) and decreased TNF (60–120 min incubation), suggesting that p-ERK is instrumental in TNF expression in LPS-stimulated primary microglia. TNF may be produced by spinal microglial following peripheral nerve injury and contribute to behavioral hypersensitivity [3]. The reduction of microglial TNF production by CBR2 activation may explain the antinociceptive effects of CBR2 agonists in rodent pain models. TNF significantly contributes to peripheral nerve injury-induced allodynia [22] and causes direct nerve sensitization [38]. Additionally, TNF induces the phosphorylation of spinal microglial MAPKs (i.e. p38) that also contributes to neuropathic pain [39,40]. This positive feedback loop between TNF and MAPKs can enhance the expression of other pro-algesic factors which may contribute to chronic pain. In contrast, the reduction of TNF expression would disrupt this positive feedback loop, reducing behavioral hypersensitivity.

**Figure 8:**
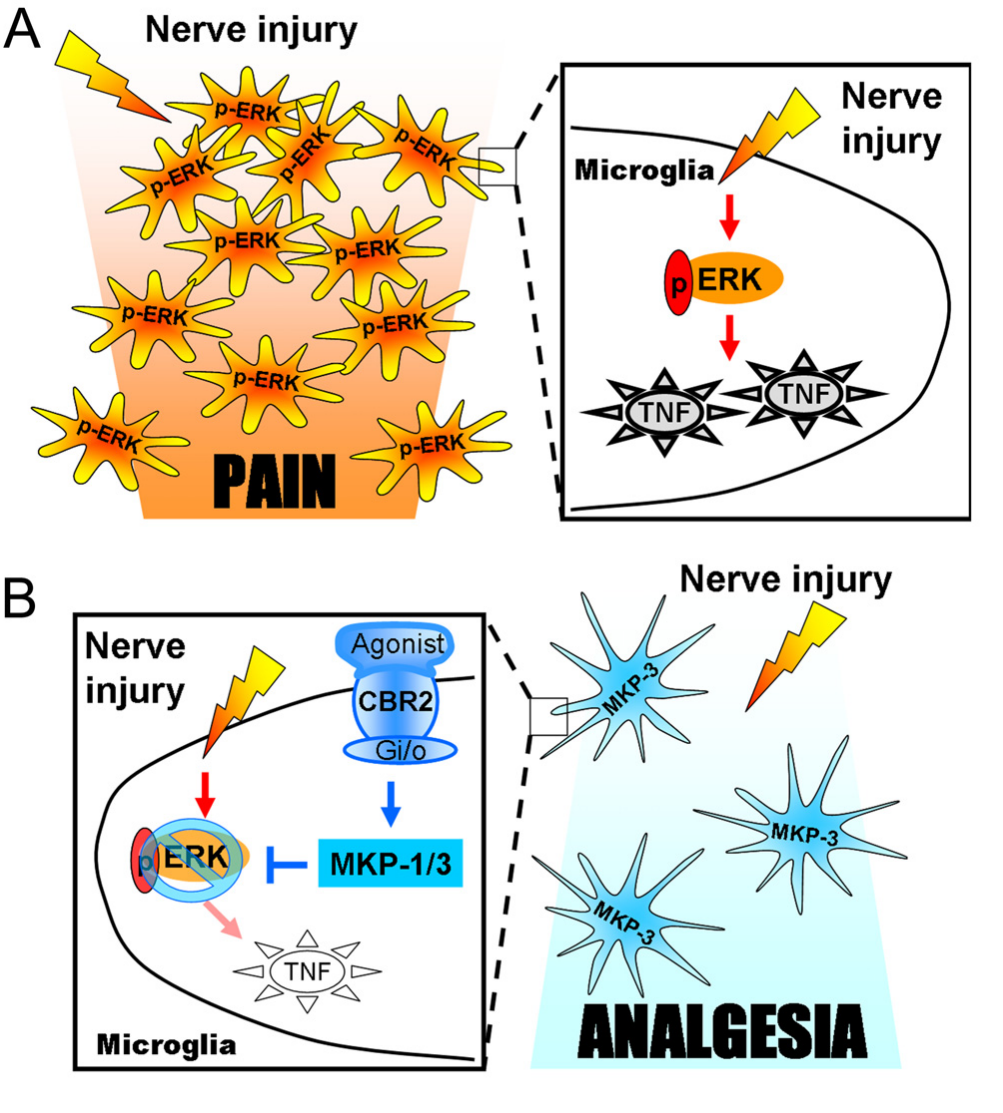
**Proposed hypotheses**. (A) Scheme of the proposed hypothesis on the mechanisms by which p-ERK induces a pro-inflammatory phenotype in microglia and contributes to neuropathic pain. Peripheral nerve injury increases microglial p-ERK in spinal cord dorsal horn, which in turn leads to the expression of TNF and an increase in microglial motility. These pro-inflammatory microglia migrate towards the spinal injured neuron area attracted by chemoattractants, such as ATP/ADP. The enhancement of pro-inflammatory microglia sensitizes spinal nociceptive neurons by increasing the concentration of pro-algesic factors, such as TNF. (B) Scheme of the proposed hypothesis on the mechanisms by which CBR2 activation-induced MKP-3 promotes an anti-inflammatory phenotype in microglia and alleviates neuropathic pain. Microglial CBR2 are increased in microglia following peripheral nerve injury. Microglial CBR2 activation induces an anti-inflammatory phenotype in microglia by increasing MKP-3 expression, which selectively inhibits p-ERK in spinal cord dorsal horn. Subsequently, ERK dephosphorylation results in a reduction of TNF expression and microglial motility. The reduction in the migration of pro-inflammatory microglia reduces the source of pro-algesic factors, such as TNF preventing neuronal sensitization and alleviating peripheral nerve injury-induced allodynia.

Cell migration is partially mediated by p-ERK [23,24]. Herein, we demonstrated that LPS-stimulated microglial motility depends on p-ERK. Additionally, the reduction of LPS-stimulated microglial migration by JWH015 was blocked by triptolide (which also inhibits MKP-3 expression). Our data strongly suggest that the modulation of the MKP-3/p-ERK pathway is the mechanism by which CBR2 activation reduces microglial migration towards ADP. ATP/ADP is one of the major chemoattractants for microglia [41], and it is released from astrocytes [42,43] or dorsal horn neurons following a pathological event, such as nerve injury [44]. Therefore, it is possible that microglial migration contributes to neuropathic pain. We hypothesize that reducing microglial p-ERK and subsequently pro-inflammatory microglial trafficking to injured neurons following nerve injury, decreases the release of pro-inflammatory mediators (such as TNF) into the synaptic milieu, which prevents neuronal sensitization, the pathological correlate to chronic pain (Figure 8).

Our findings are in contrast with previous observations. First, it has been shown that MKP-1 induction by the non-selective cannabinoids, anandamide and WIN55,212-2 resulted in ERK dephosphorylation, which in turn reduced NO, but it did not affect TNF expression in BV-2 cells. Second, CBR2 activation by endocannabinoids seems to promote chemotaxis via ERK phosphorylation in BV-2 cells [45,46]. We have demonstrated that immortalized microglial cell lines, including BV-2, may not mimic *in vivo *situations. They produce significantly lower amounts of TNF and MCP-1, and express significantly less p-ERK after LPS stimulation, and possess higher migratory rates towards ADP than primary microglia [47]. Accordingly, BV-2 and primary microglia also have different cannabinoid enzymatic profiles; i.e. primary microglia, but not BV-2 cells, express monoacylglycerol lipase [48,49]. Furthermore, contradictory findings have been reported regarding the chemoattractant effects of endocannabinoids using the microglial cell lines BV-2 or N11 [45,46,50]. Therefore, results obtained using BV-2 or other cell lines should be taken with caution until they are confirmed and directly compared with primary microglial cell experiments. The belief that endocannabinoids induce microglial chemotaxis should be revisited.

## Conclusion

Microglial reactivity is part of the pathophysiological process of several neurological diseases including neuropathic, inflammatory or postoperative pain [3,12,13,51]. We have shown that selective CBR2 activation reduces spinal glial reactivity and behavioral hypersensitivity in animal models of pain (see references above). We propose that CBR2 activation induces these effects via MKP-3 induction. In summary, our current results uncovered a cellular mechanism of action of CBR2 agonists that produces a microglial anti-inflammatory phenotype, which may modulate microglial motility *in vivo*. We identified MKP-3 and microglial migration as potential new targets for drug development. The clinical utility of CBR2 agonists is supported by their analgesic efficacy and their lack of neurological side effects in animal models of postoperative or neuropathic pain [12,13].

## Methods

### Drugs

The CBR1 antagonist AM281 (1-(2,4-Dichlorophenyl)-5-(4-iodophenyl)-4-methyl-*N*-4-morpholinyl-1*H*-pyrazole-3-carboxamide), the CBR2 antagonist AM630 (6-Iodo-2-methyl-1- [2-(4-morpholinyl)ethyl]-1*H*-indol-3-yl](4-methoxyphenyl)methanone) and the CBR2 agonist JWH015 ((2-Methyl-1-propyl-1*H*-indol-3-yl)-1-naphthalenylmethanone) were obtained from Tocris, Ellisville, MI, USA. Drugs were diluted in dimethylsulfoxide (DMSO) and then in saline to a final concentration of 100 μM (5% DMSO). The final concentration of DMSO in cell culture with cannabinoid treatments was never higher than 0.05%, and this was used as the control group (medium control). JWH015 was chosen because we have shown *in vivo *that it selectively acts on spinal microglial CBR2 in L5 nerve transection rats and reduces spinal Iba-1 expression in association with its anti-allodynic effects [13]. We chose the specific antagonists because we also showed in the same study that AM630 blocked JWH015's effects, while AM281 did not. JWH015 at 5 μM concentration has been shown to effectively reduce pro-inflammatory factors in microglia with CBR2 selectivity [16]. We chose a maximum dose of 1 μM for JWH015 (and equimolar doses for the antagonists) to assure its specificity.

Since ERKs are the only known substrates of MEK, the MEK inhibitor, UO126 (Cell Signaling, Danvers, Massachusetts, USA) was used to produce ERK inactivation. PSI2106 [52] (kindly provided by Dr. Key Brummon, University of Pittsburgh, Pennsylvania, USA), Ro-31-8220 [29] and triptolide [28,53,54] were used as MKP-1 inhibitors. UO126, PSI2106 and triptolide (Sigma, St. Louis, Missouri, USA) were diluted in DMSO and then in saline to a final concentration of 100 μM (1% DMSO). The final concentration of DMSO in cell culture with these drug treatments was never higher than 0.01%, and this was used as control group (medium control). Ro-31-8220, minocycline, adenosine diphosphate (ADP) and lipopolysaccharide (LPS, 0111:B4 serotype) were diluted in saline (Sigma).

### Cell culture

After approval by the Institutional Animal Care and Use Committee at Dartmouth College (Hanover, New Hampshire, USA), highly purified primary microglial cultures were prepared using postnatal day (P) 2–3 Harlan Sprague-Dawley pups (Indianapolis, Indiana, USA) as described previously [33,47]. Briefly, pups were decapitated and the cerebral cortices were removed; meninges were dissected away; cortical tissue was minced with a sterile scalpel blade and digested with trypsin/EDTA 1× (Mediatech, Herndon, Virginia, USA) for 15 min at 37°C. The supernatant was discarded and 5 mL Dulbecco's modified Eagle's medium (DMEM; Mediatech) supplemented with 10% charcoal-stripped fetal bovine serum (FBS; Hyclone, Logan, Utah, USA), 1.1% GlutaMax (Gibco-Invitrogen, Carlsbad, California, USA), and 1% penicillin/streptomycin (100 U/mL penicillin and 100 μg/mL streptomycin; Mediatech) containing 2000 U DNase (Sigma) were added to the tissue on ice. The tissue was triturated with a 5 mL pipette. The tissue clumps were allowed to settle and the supernatant was removed to a sterile 50 mL conical tube on ice between triturations. Triturations were repeated until no tissue clumps were observed. The final volume was diluted to 25 mL with media and centrifuged at 310 *g *for 15 min. The supernatant was discarded and the cells resuspended in media. A small aliquot of cells was stained for trypan blue (Sigma) exclusion and cells were plated at 1 × 10^6 ^cells per 75-cm^2 ^flask. Cultures were maintained at 37°C and 5% CO_2_. Media was changed every 3–4 days. After 8 days *in vitro *(DIV 8), the flasks were confluent with astrocytes and microglia. Flasks were lightly shaken by hand for 1 min and the media containing microglia was removed and centrifuged at 310 *g *for 15 min. Cultures were found to be 99% microglia by staining with OX-42 antibody (generous gift from Dr William Hickey) a marker for the CR3/CD11b receptor.

### Western blot analysis

Confluent DIV 8 primary microglia were shaken, counted and plated for at least 1.5 hr in serum free medium (SFM) at a density of 400 × 10^3 ^cells/mL. The cells were treated with different drugs (see below) using a time course incubation (15, 30, 60 and 120 min). Following treatments, the culture plates were briefly centrifuged; supernatants were removed and 60 μL of 1× Laemmli buffer (Bio-Rad, Hercules, California, USA) containing 2-mercaptoethanol (ME) (Sigma) was added to each well. Protein expression was assessed using western blot analysis. Briefly, samples and standard protein markers were subjected to sodium dodecyl sulfate-polyacrylamide gel electrophoresis (10% gels; Bio-Rad) and transferred to polyvinylidene difluoride (Bio-Rad) membranes. Non-specific binding was blocked by incubation with 5% bovine serum albumin in Tris-buffered saline-Tween 20 (0.05%; Sigma) at 22°C, then membranes were incubated overnight or for 36–40 hr at 4°C with rabbit anti-phospho-ERK 44/42 (phospho-mitogen-activated protein kinase, p-ERK, 1:500; Cell Signaling), mouse anti-total-ERK 44/42 (total mitogen-activated protein kinase, t-ERK, 1:1000; Cell Signaling), rabbit anti-MKP-1 (mitogen-activated protein kinase phosphatase-1, MKP-1, 1:400; Santa Cruz, California, USA), goat anti-MKP-3 (mitogen-activated protein kinase phosphatase-3, MKP-3, 1:400; Santa Cruz, USA) or rabbit anti-TNF (1:1000; Peprotech, Rocky Hill, New Jersey, USA). The next day (or 36–40 hr), blots were incubated for 1 h at 22°C with goat anti-rabbit, mouse or donkey anti-goat horseradish peroxidase-conjugated secondary antibodies (1:3000; Pierce, Rockford, Illinois, USA), visualized with SuperSignal West Femto Maximum Sensitivity Substrate (Pierce) for 5 min and imaged using the Syngene G-Box (Synoptics, Frederick, Maryland, USA). After incubation with the first primary and secondary antibodies, three blots were incubated for 25 min at 37°C in stripping buffer. Blots were visualized using SuperSignal West Femto Maximum Sensitivity Substrate (Pierce) for 5 min and we observed that the antibodies were completely removed. Therefore, the same stripping procedure was used to re-probe with another primary and secondary antibodies. This methodology allowed us to observe the effects of the drug treatments in different proteins from the same treated cells. Finally, blots were subsequently stripped and re-probed with mouse anti-beta-actin antibody (1:3000; Abcam, Cambridge, Massachusetts, USA), and this was used as the protein loading control. Band intensity was assessed using the analysis software package provided with the Syngene G-Box and data were quantified as relative intensity of band of interest divided by intensity of beta-actin. Normalization of p-ERK and t-ERK were also conducted against beta-actin, then p-ERK data were quantified as relative intensity of band divided by intensity of t-ERK. Data were expressed as relative intensity normalized to beta actin control and to the control group for each experiment ± SEM. Incubation of blots with only secondary antibodies did not show any protein band.

The following treatment groups were performed. Medium control (time 0) or LPS (5 ng/ml) alone groups were used as the control groups to compare the effects of selective microglial CBR2 activation on MKP-1/3 (n = 5–7), t-ERK (n = 5–7), p-ERK (n = 5–7) and TNF (n = 4) using a group with LPS + JWH015 (1 μM). To test the specificity of the CBR2 agonist (JWH015), LPS + JWH015 (1 μM) group was compared to LPS + JWH015 (1 μM) + AM281 or AM630 (1 μM, n = 9) groups. The dependence of TNF production on p-ERK has been previously shown [21,32]. To confirm this in our preparation, we used the MEK inhibitor, UO126 (1 μM, n = 6) in LPS-stimulated cells. Additionally, to probe that CBR2 activation reduces p-ERK by inducing MKP-1 and/or MKP-3, we challenged the effects of JWH015 (1 μM) in the presence of LPS with different drugs claimed to be MKP-1 inhibitors. Thus, the LPS + JWH015 (1 μM) group was compared with LPS + JWH015 (1 μM) + Ro-31-8220, triptolide or PSI2106 (1–10 μM, n = 3). All data were normalized to control groups which were given a value of 1.

### Migration

Our laboratory has previously characterized and optimized the incubation times and chemoattractants that allow optimum cell migration susceptible to pharmacological modulation [33,47]. We used Costar Transwell^® ^plates (6.5 mm diameter insert, 8.0 μm pore size, polycarbonate membrane; Corning Inc., Corning, New York, USA). For this group of experiments, the bottom chamber of these plates contained 10 μM ADP, a potent microglial chemoattractant released from injured neurons [33,41,47,55]. Confluent DIV 8 primary microglia were shaken, washed with PBS, counted using trypan blue, then placed in SFM (DMEM). Cells were resuspended at 100 × 10^3 ^cells in 200 μL SFM and treated with different drugs. To assess the effects of cannabinoid or other pharmacological treatments on primary microglia, cells were pre-treated with drugs for 2 h in the presence of LPS (5 ng/ml). Then, cells were centrifuged, medium containing the drugs was discarded and fresh SFM was added. Cells were counted using trypan blue to insure survival post-treatment (> 95% viability).

Cells were added to the top chamber (100 × 10^3 ^cells in 200 μL SFM) of a transwell plate with fibronectin-coated membranes with ADP (10 μM, 500 μL in SFM) in the bottom chamber. Cells were then allowed to migrate towards ADP for 1 or 2 h at 37°C and 5% CO_2_. Following migration, the medium in the top chamber was aspirated and the membrane gently wiped with a cotton swab to remove the cells that did not migrate. The membranes were first rinsed with PBS, the cells were then fixed with 2% formaldehyde in PBS, permeabilized with 0.01% Triton X-100 (Sigma) in PBS, and finally stained with crystal violet (Sigma). The membranes were then dried and mounted on microscope slides. Images of nine random fields (20× objective) for each membrane were captured via a Q-Fired cooled CCD camera attached to an Olympus microscope and counted by hand with aid of SigmaScan Pro imaging analysis software. Counts for all nine fields were averaged to give a mean cell count for each membrane. All experiments were completed at least three times (n = 3).

To study the effects of CBR2 activation on cell migration in LPS-stimulated microglia we used the following groups: LPS + JWH015 (0.01–1 μM, n = 9). To test the specificity of the CBR2 agonist, we challenged its effects with specific CBR1 (AM281) and CBR2 (AM630) antagonists by using the following groups: LPS + JWH015 (1 μM) + AM281 (1 μM, n = 4) or AM630 (1 μM, n = 6). To test whether p-ERK is involved in microglial migration we used a specific MEK inhibitor, UO126 (1 μM, n = 6) in LPS-stimulated cells. We also used a positive control group using LPS-stimulated microglia + minocycline (60 μM, n = 6), a dose that our laboratory has shown to be effective in reducing microglial migration in non-stimulated microglia [33]. To further test whether JWH015's effects on MKP-1/3 were causatively linked with JWH015's effects on microglial migration, we used Triptolide (10 μM, 15 min pre-treatment, n = 6) in LPS-stimulated cells + JWH015 (1 μM) incubated for 2 hr in the migration well. It has been shown that JWH015 act as chemoattractant in human monocytes when used at 20 μM concentration in parallel to an induction in ERK phosphorylation. However, JWH015 does not induce chemotaxis at 5–10 μM concentrations, [56]. Therefore, we chose 1 μM as our highest JWH015 dose tested.

### Statistical analyses

Data are expressed as mean ± SEM. Statistical analyses were completed using GraphPad Prism 4 (GraphPad Software, Inc., San Diego, California, USA). The effects of LPS and drug on proteins measured by western blot analyses and cell migration were examined using the repetitive measurements one-way analysis of variance. If significant effects were found, Dunnett's test was conducted. When appropriate (non-normally distributed), these data were evaluated using the Friedman Repeated Measures Analysis of Variance on Rank test. If significant effects were found, non-parametric Wilcoxon signed ranks tests were conducted comparing each time point to the medium control group. Between group differences were examined using two-way analysis of variance. If differences were found, Bonferroni post test was used. When appropriate (non-normally distributed), between group differences were examined at each time period using the Kruskal-Wallis test. Significant effects were followed using the Mann-Whitney U test comparing only the novel treatment to control or agonist group. Data are presented as mean ± SEM. Significance was determined at a level of *p *< 0.05.

## List of abbreviations

CBR: Cannabinoid receptor; ERK: Extracellular signal-regulated kinase; LPS: Lipopolysaccharide; MAPK: Mitogen activated protein kinases; MEK: MAP/ERK kinase; MKP: Mitogen-activated protein kinase-phosphatase; p-ERK: Phospho-extracellular signal-regulated kinase; t-ERK: Total-extracellular signal-regulated kinase; TNF: Tumor necrosis factor-α.

## Competing interests

The authors declare that they have no competing interests.

## Authors' contributions

EAR-S participated in the molecular studies, carried out the migration studies, conceived the study, performed the statistical analysis and participated in its design and coordination and drafted the manuscript. RJH participated in the cell culture preparation and migration studies, and helped to draft the manuscript. RPL participated in the cell culture preparation and molecular studies, and helped to draft the manuscript. JAD participated in the study design and coordination and edited the manuscript. All authors read and approved the final manuscript.
